# Supplementary material for: Structural basis for the complex DNA binding behavior of the plant stem cell regulator WUSCHEL
Source: Nat Commun. 2020 May 6;11:2223. doi: 10.1038/s41467-020-16024-y (PMC7203112; doi:10.1038/s41467-020-16024-y)
Supplement: Supplementary file 1 — Supplemenztary Information [file 41467_2020_16024_MOESM1_ESM.pdf]

SUPPLEMENTARY MATERIAL

Structural basis for the complex DNA binding behavior of the plant stem cell regulator WUSCHEL

Jeremy Sloan[1,+], Jana P. Hakenjos[2,#], Michael Gebert[2], Olga Ermakova[2], Andrea Gumiero[1,§,] Gunter Stier[1], Klemens Wild[1], Irmgard Sinning[1] and Jan U. Lohmann[2*]*

[1] Biochemistry Center, Heidelberg University, Im Neuenheimer Feld 328, 69120 Heidelberg, Germany

[2] Department of Stem Cell Biology, Centre for Organismal Studies, Heidelberg University, Im Neuenheimer Feld 230, 69120 Heidelberg, Germany

[+] Present address: BASF SE, Carl-Bosch-Strasse 38, 67056 Ludwigshafen, Germany

[#] Present address: Celonic AG, Eulerstrasse 55, 4051 Basel, Switzerland

[§] Present address: Instituto Poligrafico e Zecca dello Stato S.p.A., Via Salaria, I-712-00138 Roma, Italy

[*] To whom correspondence should be addressed:

Jan U. Lohmann (email: jan.lohmann@cos.uni-heidelberg.de)
Department of Stem Cell Biology
Centre for Organismal Studies, Heidelberg University,
Im Neuenheimer Feld 230, 69120 Heidelberg, Germany.

Irmgard Sinning (email: irmi.sinning@bzh.uni-heidelberg.de)
Heidelberg University Biochemistry Center (BZH)
Im Neuenheimer Feld 328, 69120 Heidelberg, Germany.

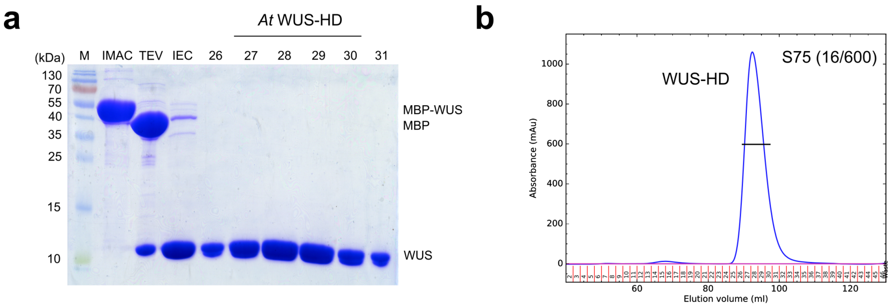

Supplementary Fig. 1 Purification and characterization of the WUS-HD

a, SDS-PAGE analysis of *At* WUS-HD purification after immobilized metal ion affinity chromatography (IMAC), TEV cleavage (TEV), ion exchange chromatography (IEC) and size-exclusion chromatography (SEC). The numbers on top of the panel correspond to peak fractions taken after gel filtration. **b,** SEC elution profile for WUS-HD. The blue line corresponds to absorbance readings at 280 nm and numbers represent collected fractions during SEC.

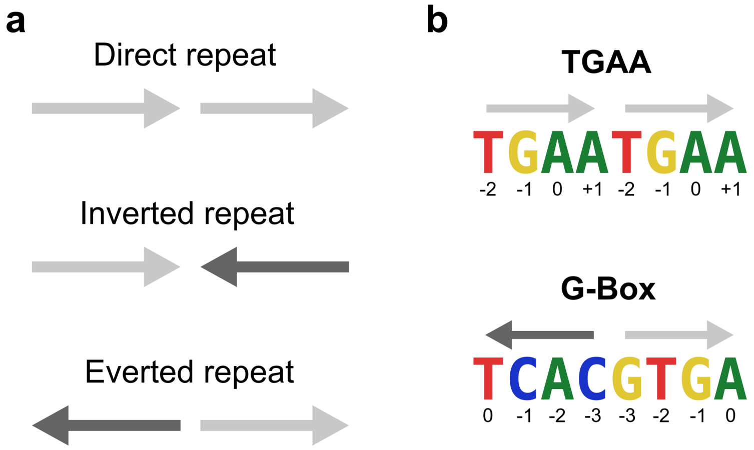

Supplementary Fig. 2 Orientation specificity of WUS DNA binding sites

a, Two DNA recognition elements can be oriented in three different configurations: direct (top), inverted (center) and everted (bottom) repeats. **b**, Direct repeat arrangement of the TGAA sequence (top) compared to the everted repeats in the G-box sequence (bottom). The recognition motif position is indicated below the sequences.

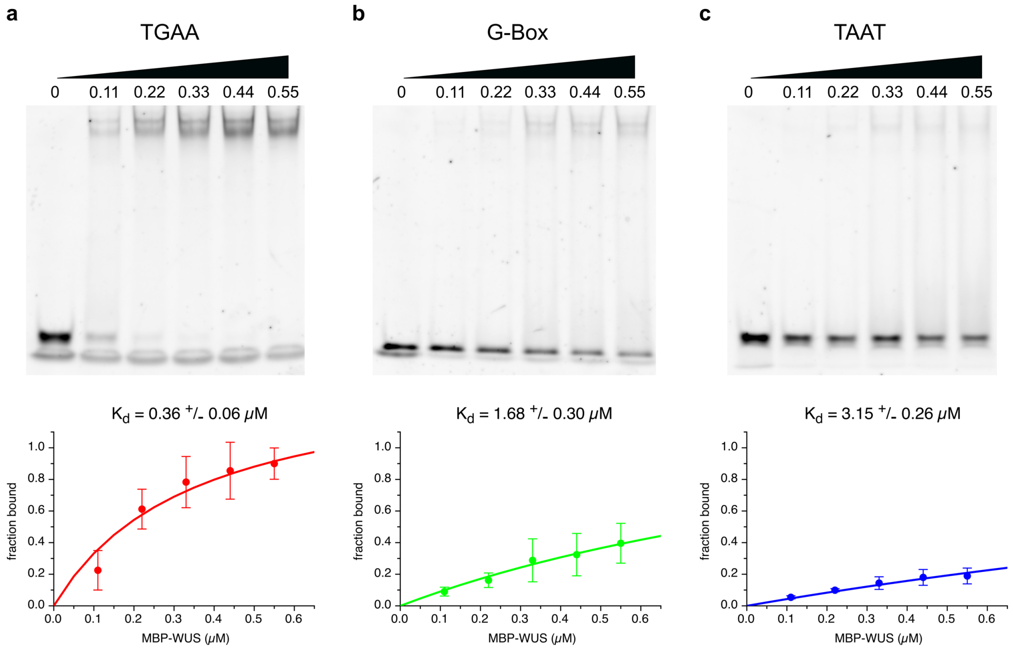

Supplementary Fig. 3 EMSAs probing the DNA-binding preferences of WUS-FL

EMSAs (top panel) and WUS-DNA saturation curves (lower panel) were performed using concentration series of WUS-FL probed with uniform levels of DNA containing TGAA (**a,** red), G-Box (**b,** green) and TAAT (**c,** blue). Protein concentrations (μM) are given on top of each gel and the calculated binding affinities are indicated below. Data are means ± SEM (error bars), n = 3. Source data are provided as a Source Data file.

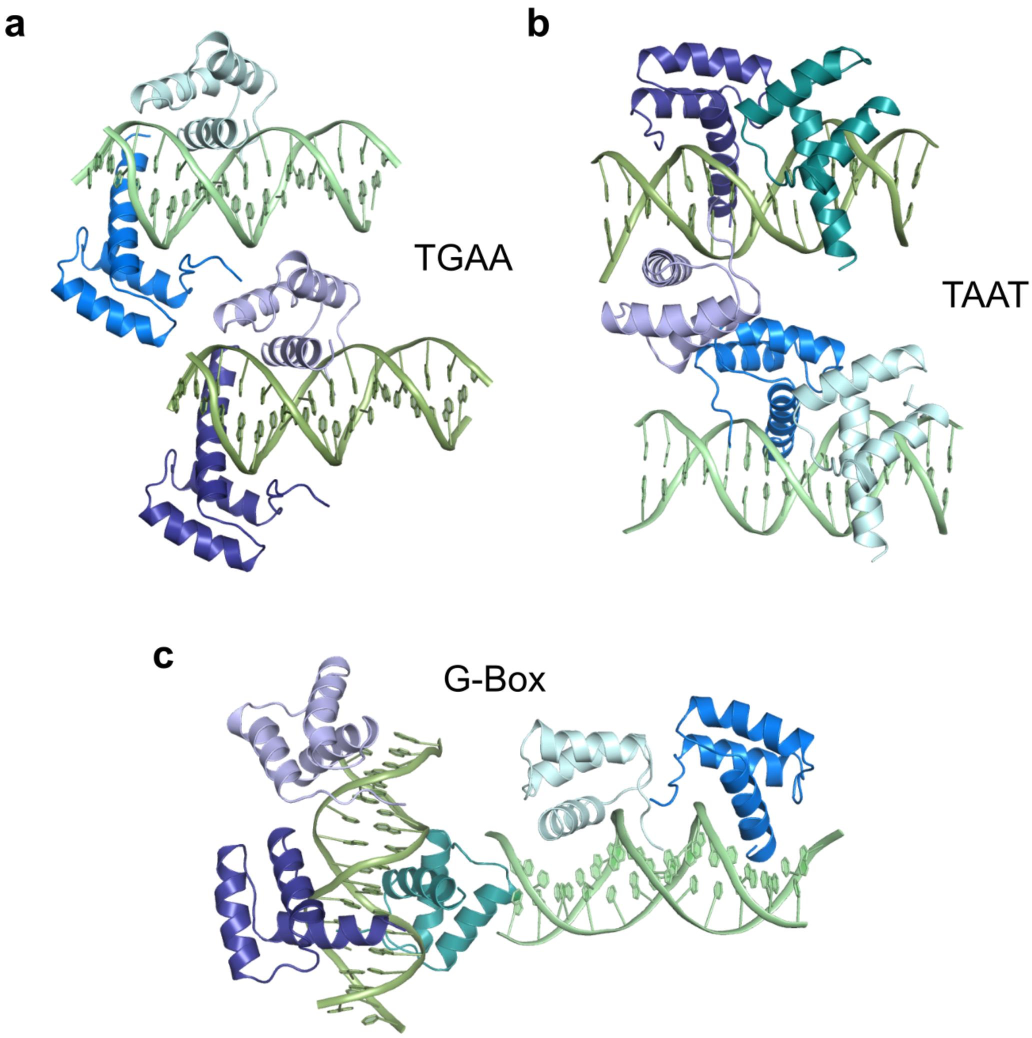

Supplementary Fig. 4 Crystal packing of the TGAA, TAAT and G-Box structures

a, The asymmetric unit of the TGAA structure contains two complexes consisting of two DNA molecules and four WUS-HD molecules. **b**, The asymmetric unit of the TAAT structure contains two complexes consisting of two DNA molecules and five WUS-HD molecules. **c**, The asymmetric unit of the G-Box structure contains two complexes consisting of two DNA molecules and five WUS-HD molecules. Colors schemes are related to Fig. 2, with DNA in shades of green and WUS-HD in shades of blue, and one complex is shown in lighter colors.

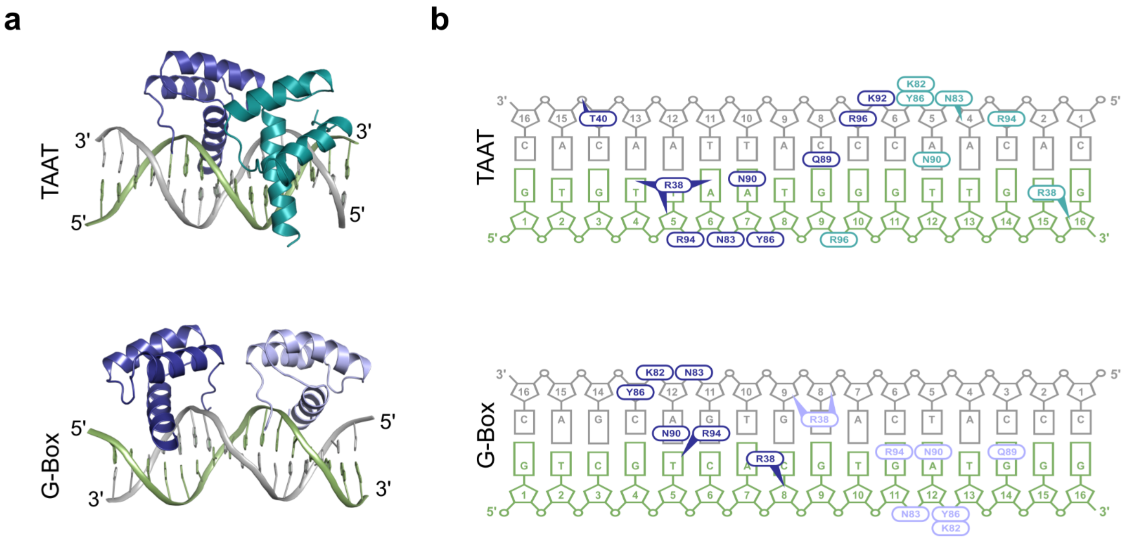

Supplementary Fig. 5 Additional HD binding in the TAAT and G-Box structures

a, Overall structures of WUS-DNA complexes showing two WUS molecules per DNA for TAAT (top) and G-Box (bottom). Colors of protein/DNA are the same as in Fig. 2. **b**, Summary of WUS-DNA interactions including the additional HD molecule. Order and colors are the same as in A and numbering and symbols are according to Fig. 2.

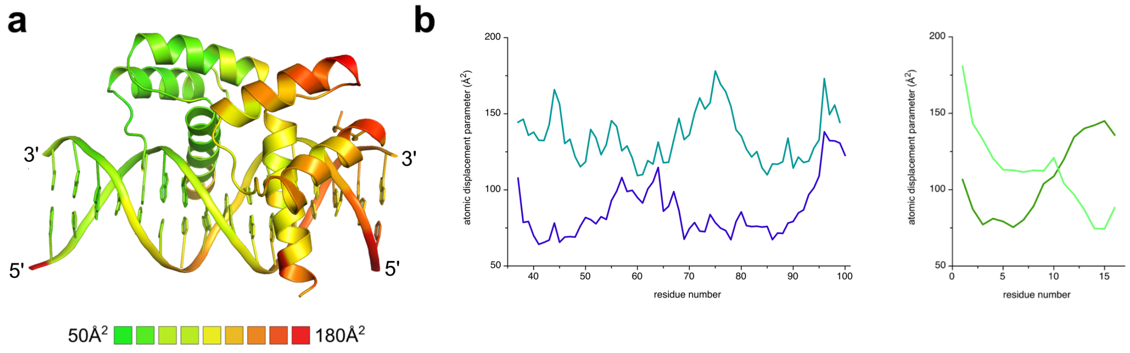

Supplementary Fig. 6 B-factor plot for the WUS-TAAT structure

a, Structure of WUS-HD bound to TAAT DNA colored according to residue average B-factor, scaled from 50 (green) to 180 (red) Å^2. **b**, Atomic displacement parameters plotted against the residue number of WUS-HD (left) and the TAAT DNA probe (right). Colors and numbering are the same as in **Error! Reference source not found.**.

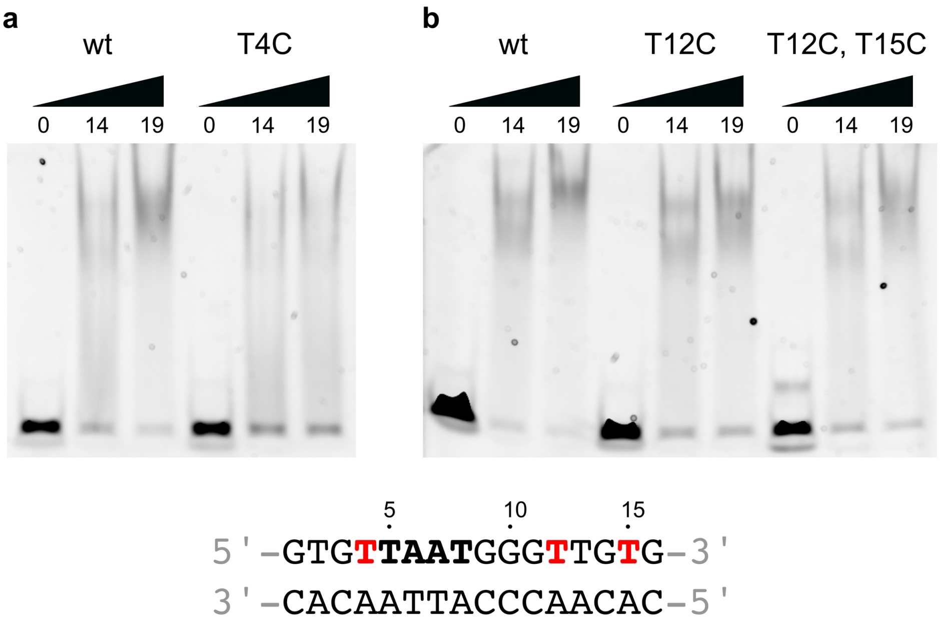

Supplementary Fig. 7 EMSAs probing WUS-HD configuration on the TAAT probe

Altered DNA-binding behavior of WUS-HD to a TAAT motif with specific mutations in flanking regions associated with additional protein-DNA contacts. The respective point mutations T4C (**a**), T12C and T15C (**b**) are highlighted in the sequence (red) at the bottom and the protein concentration (µM) is indicated at the top.

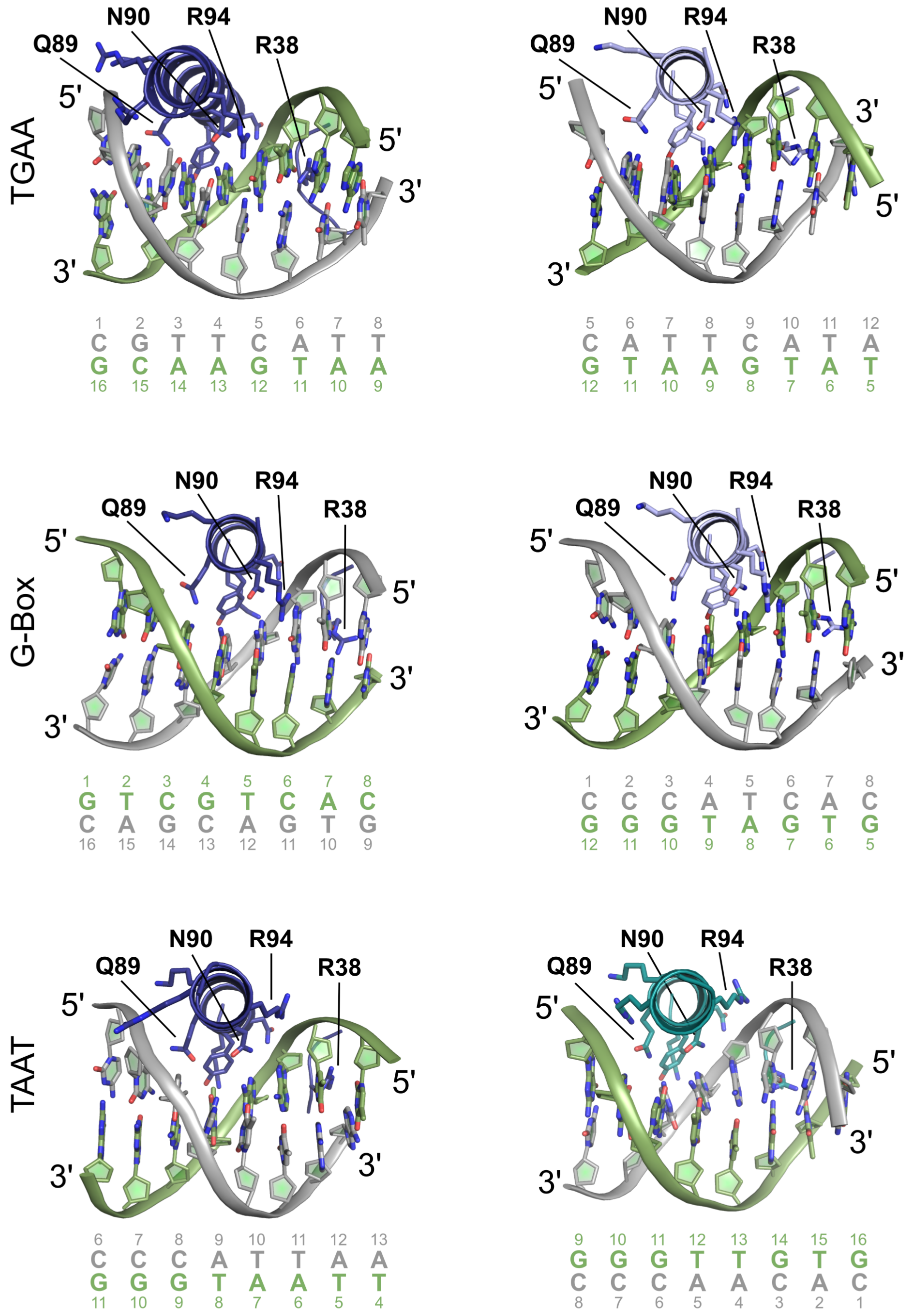

Supplementary Fig. 8 Detailed DNA interactions of WUS-HD

Detailed comparison of the DNA-contacting residues of each WUS-HD bound to TGAA (top), G-Box (center) and TAAT (bottom). Only the helix α3 and the N-terminal arm of WUS-HD involved in major-groove and minor-groove interactions, respectively, are shown for clarity. Colors and numbering of DNA bases are the same as in **Error! Reference source not found.**.

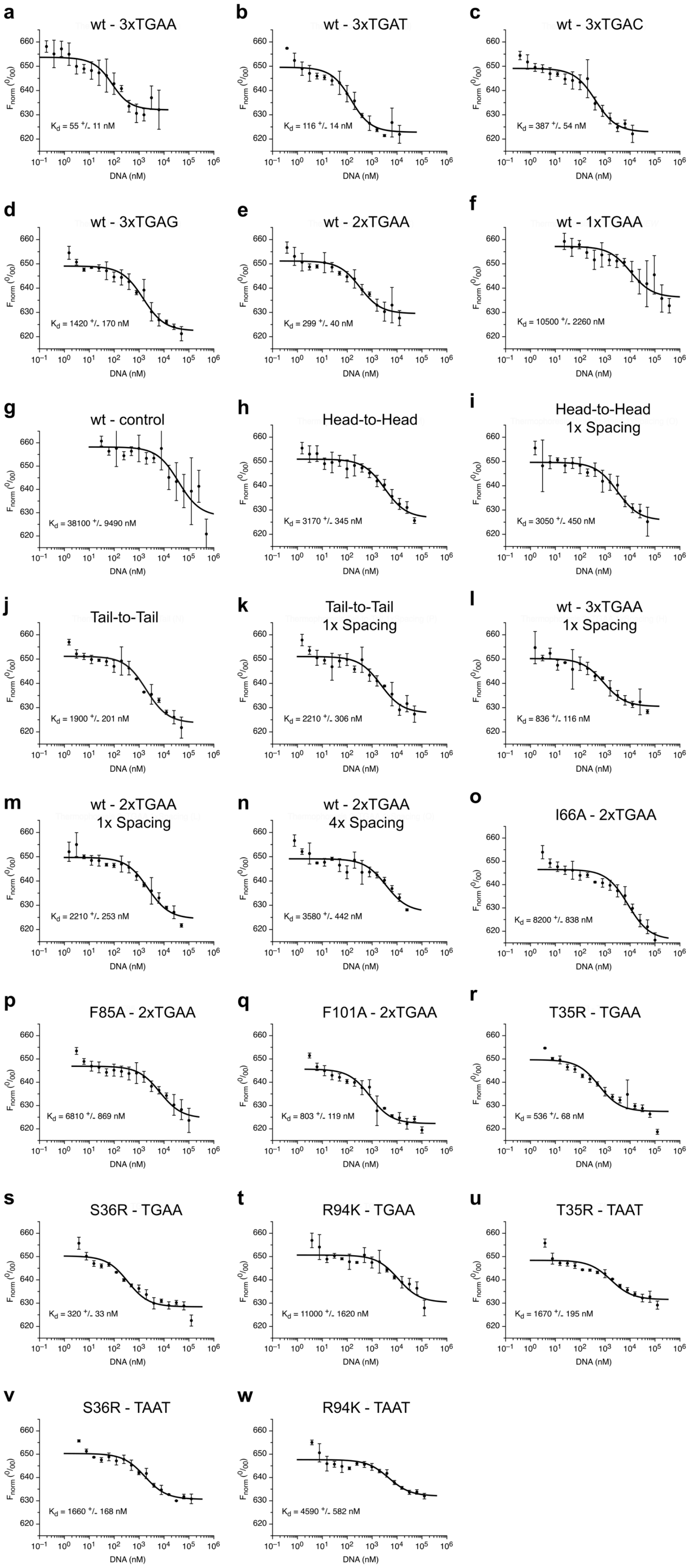

a
wt - 3xTGAA
K_d = 55 +/- 11 nM
b
wt - 3xTGAT
K_d = 116 +/- 14 nM
c
wt - 3xTGAC
K_d = 387 +/- 54 nM
d
wt - 3xTGAG
K_d = 1420 +/- 170 nM
e
wt - 2xTGAA
K_d = 299 +/- 40 nM
f
wt - 1xTGAA
K_d = 10500 +/- 2260 nM
g
wt - control
K_d = 38100 +/- 9490 nM
h
Head-to-Head
K_d = 3170 +/- 345 nM
i
Head-to-Head
1x Spacing
K_d = 3050 +/- 450 nM
j
Tail-to-Tail
K_d = 1900 +/- 201 nM
k
Tail-to-Tail
1x Spacing
K_d = 2210 +/- 306 nM
l
wt - 3xTGAA
1x Spacing
K_d = 836 +/- 116 nM
m
wt - 2xTGAA
1x Spacing
K_d = 2210 +/- 253 nM
n
wt - 2xTGAA
4x Spacing
K_d = 3580 +/- 442 nM
o
I66A - 2xTGAA
K_d = 8200 +/- 838 nM
p
F85A - 2xTGAA
K_d = 6810 +/- 869 nM
q
F101A - 2xTGAA
K_d = 803 +/- 119 nM
r
T35R - TGAA
K_d = 536 +/- 68 nM
s
S36R - TGAA
K_d = 320 +/- 33 nM
t
R94K - TGAA
K_d = 11000 +/- 1620 nM
u
T35R - TAAT
K_d = 1670 +/- 195 nM
v
S36R - TAAT
K_d = 1660 +/- 168 nM
w
R94K - TAAT
K_d = 4590 +/- 582 nM
F_norm (0/00)
DNA (nM)

Supplementary Fig. 9 Summary of MST measurements

DNA-binding affinity of YFP-WUS wild-type determined by MST for (**a**) 3xTGAA, (**b**) 3xTGAT, (**c**) 3xTGAC, (**d**) 3xTGAG, (**e**) 2xTGAA, (**f**) 1xTGAA, (**g**) control without TGAA, (**h**) Head-to-Head arrangement of TGAA, (**i**) Head-to-Head arrangement of TGAA with 1x nucleotide spacer, (**j**) Tail-to-Tail arrangement of TGAA, (**k**) Tail-to-Tail arrangement of TGAA with 1x nucleotide spacer, (**l**) 3xTGAA with 1x nucleotide spacer, (**m**) 2xTGAA with 1x nucleotide spacer and (**n**) 2xTGAA with 4x nucleotide spacer. Binding affinity for 2xTGAA DNA of YFP-WUS with (**o**) I66A, (**p**) F85A and (**q**) F101A mutation. Binding affinity for TGAA DNA of YFP-WUS with (**r**) T35R, (**s**) S36R and (**t**) R94K mutation. Binding affinity for TAAT DNA of YFP-WUS with (**u**) T35R, (**v**) S36R and (**w**) R94K mutation. Data are means ± SEM (error bars), n = 3. Source data are provided as a Source Data file.

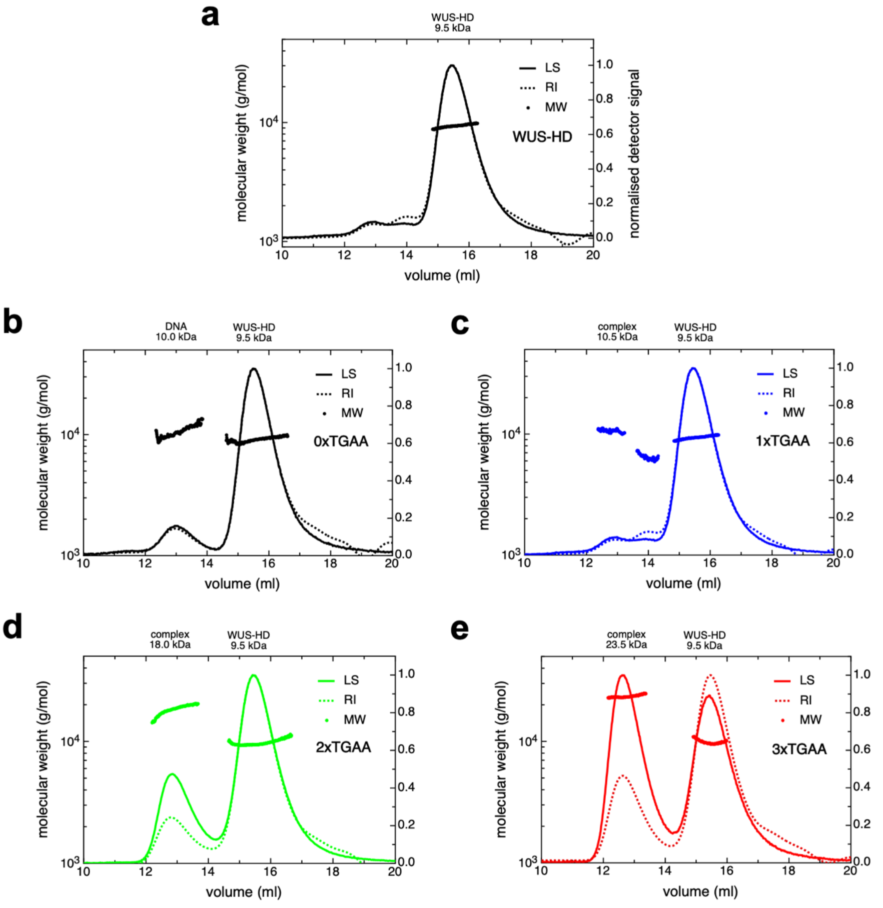

Supplementary Fig. 10 MALS analysis of the WUS-DNA complex

SEC-MALS analysis of WUS-HD (**a**) and in the presence of control DNA (**b**), 1xTGAA (**c**), 2xTGAA (**d**) and 3xTGAA (**e**). The colored lines correspond to light scattering (LS) readings and the dashed lines correspond to the refractive index (RI) readings. Molar mass (MW) distributions across the peaks are shown as dots. The average calculated molecular weight for each peak is given on top of each panel. Source data are provided as a Source Data file.

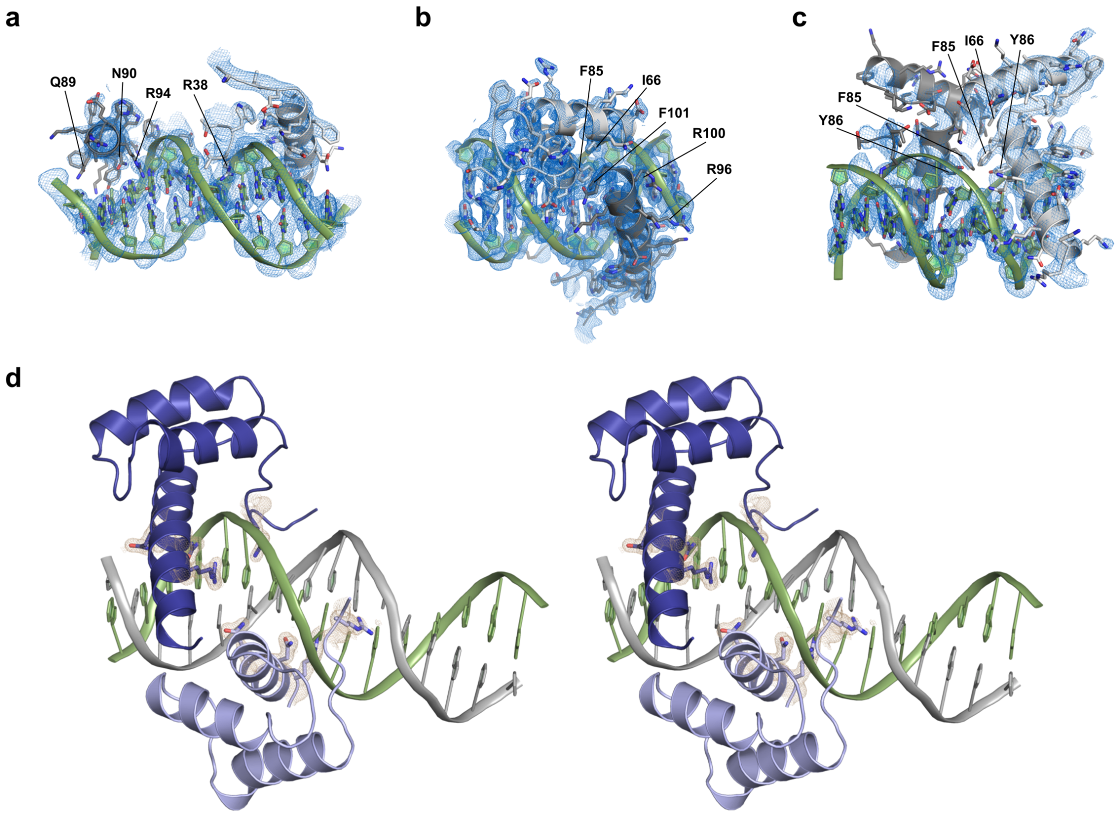

Supplementary Fig. 11 Electron density map of selected regions of WUS-DNA interactions

Close-up view of the WUS-DNA interaction highlighting DNA base-recognition in the G-Box complex (**a**) and showing the WUS dimerization interface in the TGAA (**b**) and TAAT complex (**c**). DNA is shown in green and WUS-HDs are depicted in white and gray. Relevant residues of WUS are indicated and the 2Fo-Fc electron density maps (blue mesh) are contoured at 1.0 σ. **d**, Stereo view of WUS-HD binding to TGAA DNA. The electron density represents a 2Fo-Fc map (orange), contoured at 1.0 σ and displayed with a radius of 2.0 Å around the residues involved in base-specific contacts. Colors of protein/DNA are the same as in **Error! Reference source not found.**.

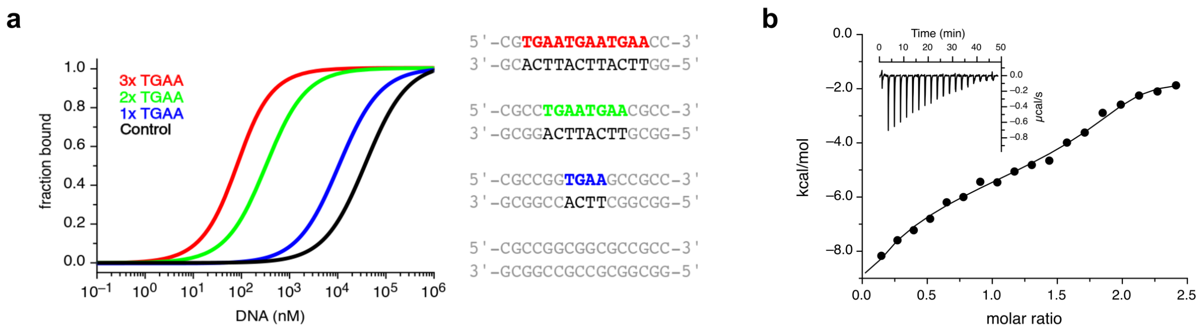

Supplementary Fig. 12 Binding stoichiometry of the WUS-DNA interaction

a, MST-analysis of WUS DNA-binding cooperativity. The number of recognition motifs is indicated: 3xTGAA (red), 2xTGAA (green), 1xTGAA (blue) and a control without TGAA (black). **b**, Quantification of the interaction between WUS-HD and 2xTGAA DNA by isothermal titration calorimetry (ITC).

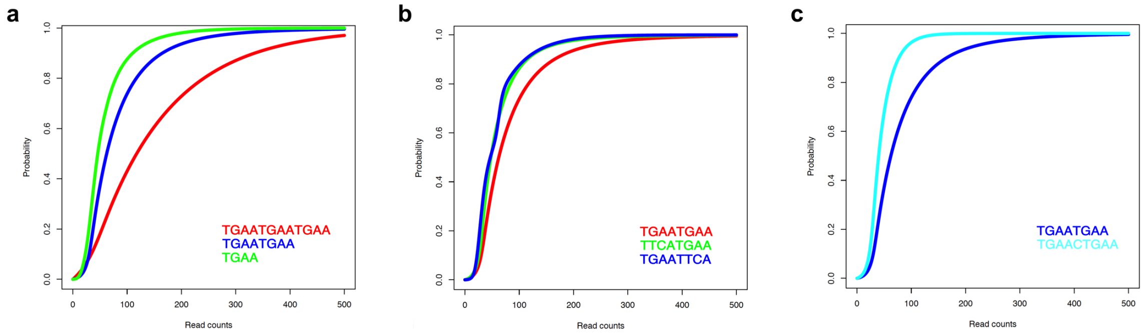

Supplementary Fig. 13 Analysis of WUS chromatin binding *in vivo* by ChIP-seq

a, Binding probabilities of WUS for 3xTGAA (red), 2xTGAA (blue) and 1xTGAA (green). **b**, Binding probabilities of WUS for 2xTGAA (red), Head-to-Head (green) and Tail-to-Tail (blue). **c**, Binding probabilities of WUS for 2xTGAA (blue) and 2xTGAA with 1xSpacing (light blue). Curves shifted to the right indicate a higher probability of a given sequence element to be associated with chromatin exhibiting high WUS occupancy and hence high affinity binding.

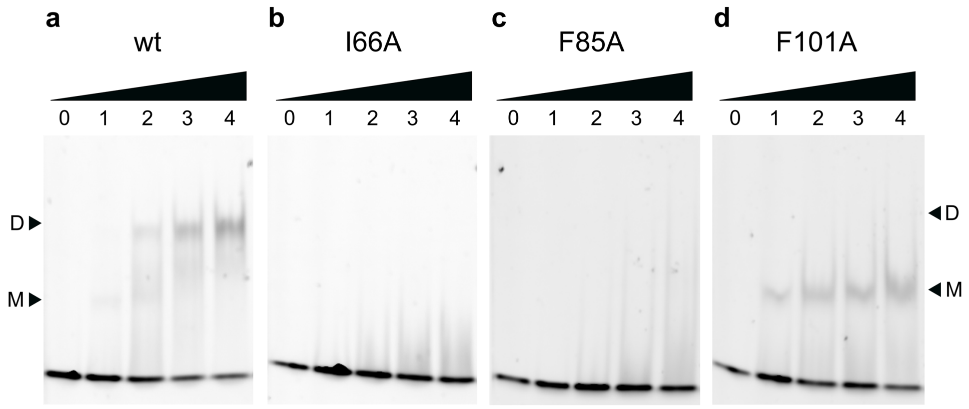

Supplementary Fig. 14 EMSAs probing the WUS dimerization interface.

Altered DNA-binding behavior to a 2xTGAA motif of WUS-HD wt (**a**), I66A (**b**), F85A (**c**) and F101 (**d**). Monomer (M) and dimer (D) bound forms of WUS are indicated and the protein concentration (µM) is given at the top.

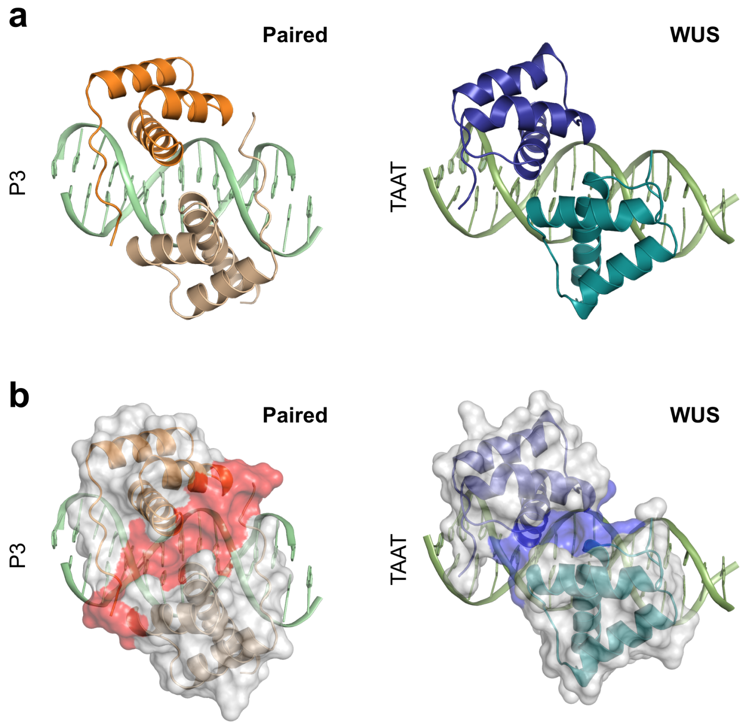

Supplementary Fig. 15 Comparison of the HD dimer between Paired and WUS

a, Side-by-side comparison of the HD homodimers of Paired bound to P3 DNA sequence (left, PDB 1FJL[45]) and WUS bound to the TAAT DNA sequence (right). **b**, Same view as before now with surface representation (grey), highlighting the interaction interface formed between the two HD molecules of Paired (red) and WUS (blue).

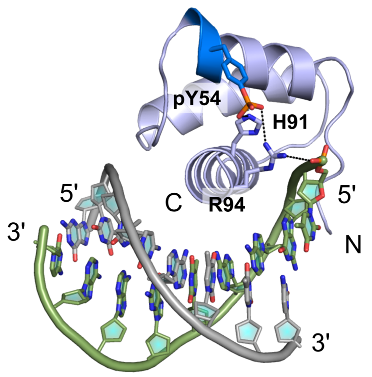

Supplementary Fig. 16 Structural model of Y54 phosphorylation

Model of WUS-DNA complex with phosphorylated tyrosine (pY54) highlighting the hydrogen bond network of the phosphate group of pY54 from the π-helix (blue). Relevant residues of WUS involved in the interaction are indicated. WUS-HD is depicted in light blue and DNA-strands are shown in grey and green.

Supplementary Table 1. Oligonucleotides used for cloning and mutagenesis

Oligomer name	Oligomer sequence
Fwd_*At* WUS HD	GCTTCCATGGGTCAGACCAGCACGAGGTGGAC
Rev_*At* WUS HD	GCTTCTCGAGTTAGGATCCTCCGTTGAATCTCTTCT TCTGACG
Fwd_*At* WUS	ATTCGGATCCATGGAGCCGCCACAGCAT
Rev_At WUS	TGTGCTCGAGCTAGTTCAGACGTAGCTC
Fwd_*At* WUS HD I66A	GATCAGGCGCAGAAGATCACTGCAAGGCTG
Rev_*At* WUS HD I66A	CTTCTGCGCCTGATCGGCTGTTGGTGACC
Fwd_*At* WUS HD F85A	GAACGTCGCGTACTGGTTCCAGAACCATAAGGC
Rev_*At* WUS HD F85A	CAGTACGCGACGTTCTTGCCCTCAATCTTTC
Fwd_*At* WUS HD F101A	GAAGAGAGCGAACGGAGGATCCTAACTCGAGTG
Rev_*At* WUS HD F101A	CCGTTCGCTCTCTTCTTCTGACGCTCACGAG
Fwd_*At* WUS HD T35R	TGTCGCCAGAGGAGCACGAGGTGGA
Rev_*At* WUS HD T35R	TCCACCTCGTGCTCCTCTGGCGACA
Fwd_*At* WUS HD S36R	TGTCGCCAGACCAGGACGAGGTGGA
Rev_*At* WUS HD S36R	TCCACCTCGTCCTGGTCTGGCGACA
Fwd_*At* WUS HD R94K	CCATAAGGCTAAGGAGCGTCAGA
Rev_*At* WUS HD R94K	TCTGACGCTCCTTAGCCTTATGG
Fwd_*At* WUS HD RRK	TGTCGCCAGAGGAGGACGAGGTGGA
Rev_*At* WUS HD RRK	TCCACCTCGTCCTCCTCTGGCGACA

Supplementary Table 2. Oligonucleotides used for crystallization

Oligomer name	Oligomer sequence
TGAA fwd	AGTGTATGAATGAACG
TGAA rev	CGTTCATTCATACACT
G-Box fwd	GTCGTCACGTGATGGG
G-Box rev	CCCATCACGTGACGAC
TAAT fwd	GTGTTAATGGGTTGTG
TAAT rev	CACAACCCATTAACAC

Supplementary Table 3. Oligonucleotides used for MST

Oligomer name	Oligomer sequence
3xTGAA fwd	CGTGAATGAATGAACC
3xTGAA rev	GGTTCATTCATTCACG
2xTGAA fwd	CGCCTGAATGAACGCC
2xTGAA rev	GGCGTTCATTCAGGCG
1xTGAA fwd	CGCCGGTGAAGCCGCC
1xTGAA rev	GGCGGCTTCACCGGCG
Negative Control fwd	CGCCGGCGGCGCCGCC
Negative Control rev	GGCGGCGCCGCCGGCG
3xTGAT fwd	CGTGATTGATTGATCC
3xTGAT rev	GGATCAATCAATCACG
3xTGAC fwd	CGTGACTGACTGACCC
3xTGAC rev	GGGTCAGTCAGTCACG
3xTGAG fwd	CGTGAGTGAGTGAGCC
3xTGAG rev	GGCTCACTCACTCACG
TGAA Head-to-Head fwd	CGCCTTCATGAACGCC
TGAA Head-to-Head rev	GGCGTTCATGAAGGCG
TGAA Head-to-Head 1xSpacing fwd	CGCTTCACTGAACGCC
TGAA Head-to-Head 1xSpacing rev	GGCGTTCAGTGAAGCG
TGAA Tail-to-Tail fwd	CGCCTGAATTCACGCC
TGAA Tail-to-Tail rev	GGCGTGAATTCAGGCG
TGAA Tail-to-Tail 1xSpacing fwd	CGCTGAACTTCACGCC

TGAA Tail-to-Tail 1xSpacing rev	GGCGTGAAGTTCAGCG
3xTGAA 1xSpacing fwd	CTGAACTGAACTGAAC
3xTGAA 1xSpacing rev	GTTCAGTTCAGTTCAG
2xTGAA 1xSpacing fwd	CGCTGAACTGAACGCC
2xTGAA 1xSpacing rev	GGCGTTCAGTTCAGCG
2xTGAA 4xSpacing fwd	CGTGAAGCGCTGAACC
2xTGAA 4xSpacing rev	GGTTCAGCGCTTCACG

Supplementary Table 4. Oligonucleotides used for fluorescent EMSAs

Oligomer name	Oligomer sequence
f_TGAA fwd	CATCGTCGTTCATTCAATGGG
f_TGAA rev	CCCATTGAATGAACGACGATG
f_G-Box fwd	CATCGTCGTCACGTGATGGG
f_G-Box rev	CCCATCACGTGACGACGATG
f_TAAT fwd	CAATGTGTTAATGGGTTGTT
f_TAAT rev	AACAACCCATTAACACATTG
f_TAAT wt fwd	GTGTTAATGGGTTGTG
f_TAAT wt rev	CACAACCCATTAACAC
f_TAAT T4C fwd	GTGCTAATGGGTTGTG
f_TAAT T4C rev	CACAACCCATTAGCAC
f_TAAT T12C fwd	GTGTTAATGGGCTGTG
f_TAAT T12C rev	CACAGCCCATTAACAC
f_TAAT T12C, T15C fwd	GTGTTAATGGGCTGCG
f_TAAT T12C, T15C rev	CGCAGCCCATTAACAC
