# Supplementary material for: Structural basis for the complex DNA binding behavior of the plant stem cell regulator WUSCHEL
Source: Nat Commun. 2020 May 6;11:2223. doi: 10.1038/s41467-020-16024-y (PMC7203112; doi:10.1038/s41467-020-16024-y)
Supplement: Supplementary file 3 — Description of Additional Supplementary Files [file 41467_2020_16024_MOESM3_ESM.docx]

**Description of Additional Supplementary Files**

**File name:** Supplementary Dataset 1

**Description:** Supplementary information on ChIP-seq based affinity measurements, including figures, code and interactive tables
