# Supplementary material for: Structural basis for the complex DNA binding behavior of the plant stem cell regulator WUSCHEL
Source: Nat Commun. 2020 May 6;11:2223. doi: 10.1038/s41467-020-16024-y (PMC7203112; doi:10.1038/s41467-020-16024-y)
Supplement: Supplementary file 4 — Supplementary Data 1 [file 41467_2020_16024_MOESM4_ESM.zip › 213982_2_data_set_4534564_q8d416.html]

Structural basis for the complex DNA binding behavior of the plant stem cell regulator WUSCHEL


# Structural basis for the complex DNA binding behavior of the plant stem cell regulator WUSCHEL

#### ChIP-seq based TGAATGAA, TCACGTGA and TTAATGG affinity measure

#### Jeremy Sloan, Jana P. Hakenjos, Michael Gebert, Olga Ermakova, Andrea Gumiero, Gunter Stier, Klemens Wild, Irmgard Sinning and Jan U. Lohmann

## R Markdown

This is an R Markdown document. It allows you to get a copy of each data table by using copy button and pasting into any document (Excel, Word, txt).

## Bioinformatic methods

We analyzed ChIP-seq data from Arabidopsis seedlings with ectopically induced WUS expression (ref.28) to assess the affinity of native WUS protein to chromatin containing the three binding motifs studied here (TGAATGAA, TCACGTGA, TTAATGG). As a proxy for affinity, the number of reads aligned to regions containing the corresponding k-mer and simultaneously belonging to known WUS peaks (ref.28) was used. For this purpose, for each k-mer of interest, genomic coordinates (chromosome, start, end) were extracted from TAIR10 genome and corresponding BED files were created. Then these regions were intersected with known WUSCHEL binding sites from ref.28 using R-package GenomicRanges v1.32.6 (ref.67) and a window of \(\pm\) 25 bp around the intersection-sequence coordinates was defined. The resulting genomic intervals were transformed into annotation file (GTF) and used for counting aligned reads from WUS ChIP-seq data (GEO accession GSE122611) with featureCounts v1.6.3 (ref. 68). The obtained counts per k-mer regions were visualized by empirical cumulative distribution using ecdf function in R and smoothed using plogspline function from R-package logspline v2.1.15 (ref. 69).

## Annotation files for counting reads

## Read count tables

## Empirical cumulative distribution function of read counts
